# Why, Who, When, and How? Rationale for Considering Allogeneic Stem Cell Transplantation in Children with Sickle Cell Disease

**DOI:** 10.3390/jcm8101523

**Published:** 2019-09-22

**Authors:** Françoise Bernaudin

**Affiliations:** French Referral Center for Sickle Cell Disease; SFGM-TC (Société Française de Greffe de Moelle et de Thérapie Cellulaire); DrepaGreffe Association 20 rue de Coulmiers, 94130 Nogent sur Marne, France; francoise.bernaudin@chicreteil.fr

**Keywords:** sickle cell disease, sickle cell anemia, hematopoïetic stem cell transplantation, transfusion, hydroxyurea, cerebral vasculopathy, vaso-occlusive crisis, acute-chest syndrome, stroke, cerebral silent infarct

## Abstract

Considering the progress made in the management of sickle cell disease during the past 30 years, along with the excellent results obtained with hematopoietic stem cell transplantation (SCT), it is important to reexamine why, who, when and how to recommend allogeneic SCT in children with sickle cell disease. While sickle cell disease has a low risk of death in children and a high risk for morbidity during aging, SCT carries an early risk of death, graft-vs-host disease and infertility. Nevertheless, SCT offers at least 95% chance of cure with low risk of chronic graft-vs-host disease when a matched-sibling donor is available and the risks of infertility can be reduced by ovarian, sperm or testis cryopreservation. Thus, all available therapies such as hydroxyurea, transfusions and SCT should be presented to the parents, providers, and affected children and discussed with them from infancy. Furthermore, the use of these therapies should be adjusted to the severity of the disease and to local availabilities in order to choose the treatment offering the best benefit/risk ratio.

## 1. Introduction

Sickle cell disease (SCD) is an increasing global health problem, as it is estimated that each year, 300,000 infants will be born with the homozygous sickle mutation [Glu6Val, rs334] in the β-globin gene, and this number could actually rise to 400,000 by 2050 [1]. Even in the US and Europe, sickle cell disease is the most common monogenic disorder, but the vast majority of these births occur in Nigeria, the Democratic Republic of Congo and India. The most severe forms, i.e., homozygous HbSS and HbS/β0-thalassemia in which HbA is not produced, are referred as sickle-cell anemia (SCA), while the two other forms of SCD, i.e., the compound heterozygous HbSC and the HbS/βthalassemia exhibit lower severity.

Upon deoxygenation, the abnormal HbS polymerizes, tranforming the normally flexible red cell shape into an elongated rigid form, which leads to vaso-occlusion and pain crisis. Repeated ischemia-reperfusion episodes are responsible for many of the acute and chronic complications affecting all major organs (anemia, hemolysis, acute splenic sequestration, stroke, cerebral silent infarcts, cognitive impairment, retinopathy, avascular osteonecrosis, leg ulcers, priapism, proteinuria, renal failure, cholelithiasis, hepato-cholangiopathy, pulmonary hypertension, etc.) [2,3].

## 2. Why Consider Allogeneic Stem Cell Transplantation in Children with Sickle Cell Disease?

In high-income countries, many advances have been made in the management of SCD in the last 30 years, such as pneumococcal prevention [4,5], introduction of hydroxyurea [6], exchange transfusions [7], oral iron chelation [8], and early detection of cerebral vasculopathy [9,10]. However, over time, the outcome for matched-sibling donor stem cell transplantation (MSD-SCT) has also significantly improved [11,12,13,14,15,16,17], to such extent that SCT indication has more recently been extended to adults [18,19,20] and to patients with no MSD using haplo-relative [21] or unrelated donor transplantation [22,23]. Moreover, the risk of infertility post-SCT may be decreased by pre-transplant ovarian [24], sperm or testis cryopreservation [25] in myeloablative SCT or by using non-myeloablative SCT [18,19,20]. While the risk of death before the age of 18 has been drastically decreased to less than 5% in Europe and the US [26,27,28,29], deaths still occur early in adults with no improvement of the median age at death, which was 42 years for males and 48 years for females in 1994 [30] versus 38 years for males and 42 years for females in 2013 [31]. Thus, while the pediatric mortality rate has decreased by 3% each year, it has increased in adults by 1% each year during the same period [31]. Moreover, significant progressive morbidity has been observed observed during aging in SCD-patients [31].

However, treatment and management of the disease remain costly and non-accessible to patients living in sub-Saharan Africa where the risk of death before the age of five years can be as high as 90% [32].

### 2.1. Non-Transplant Intensive Therapies in Sickle Cell Disease Patients

#### 2.1.1. Hydroxyurea

Hydroxyurea treatment has been the first drug approved by the Federal Drug Administration in the US for adult SCD patients in 1998, while it was approved for children in 2007 in Europe and in 2017 in the US. HbF and hemoglobin levels are known to be increased by hydroxyurea, while leukocytes, neutrophils, platelets, reticulocytes, LDH are decreased [33]. Thus, blood rheology is improved, hemolysis is decreased and a randomized trial in 1995 [6] showed that hydroxyurea allowed to significantly reduce the rate of vaso-occlusive crises (VOC), acute chest syndromes (ACS) and the need for transfusion in adult patients. The same results were thereafter obtained in children [34,35,36]. A significant association between high HbF levels and better survival has been shown [30] and several studies reported better survival on hydroxyurea treatment [37,38,39,40]. However, the response level was dependent on the initial HbF level and on beta haplotypes. BEN/BEN SCA patients have the best response to hydroxyurea, possibly due to the higher prevalence of the favorable *BCL11A/*rs1427407 T allele, while CAR/CAR have the lowest response [41]. Moreover, despite a significant reduction of crises and improved survival, hydroxyurea treatment did not modify several markers of organ damage such as glomerular filtration and tricuspid regurgitation jet velocity (TRJV) [40,42] and did not prevent silent infarcts, suggesting that organ damage still occurs on hydroxyurea [43]. Moreover, hydroxyurea is associated with higher HbF and hemoglobin, but also higner erythropoietin level, suggesting relatively lower oxygen delivery [42]. Another issue with long-term hydroxyurea treatment is the risk of infertility in men and data are still lacking regarding males who received hydroxyurea during infancy [44]. While the recommendations in the US are to systematically introduce hydroxyurea as soon as nine months of age [45], in other countries such as France, recommendations are to keep patients under observation, and to only introduce hydroxyurea when symptoms related to sickle cell anemia are present and a first complete check-up is performed in the 2nd year of life. 

#### 2.1.2. Other Drugs

Several randomized trials have evaluated the efficiency of three other drugs. Oral glutamine was recently approved by the FDA. It has been shown to reduce the crisis rate, but did not improve hemolysis [46]. Moreover, the main study had methodological limitations and the effect of glutamine on painful episodes was modest compared to hydroxyurea. Crizanlizumab administered monthly and intravenously, significantly reduced the crisis rate, but hemolysis was also not reduced [47]. Voxelator (GBT 440), a modulator of hemoglobin oxygen affinity, has been shown very recently to inhibit Hb polymerization, and decrease hemolysis without an enhancing the EPO levels [48], suggesting normal oxygen delivery.

#### 2.1.3. Transfusion

Blood transfusion allows not only to correct anemia and to dilute sickle cells, but to reverse the polymerization, principally by local oxygenation via the import of deformable normal red blood cells to the location of vascular obstruction. Thus, transfusion has been the first major treatment for sickle cell patients that could control vaso-occlusive crisis and acute chest syndrome, limit the consequences of stroke, and prevent stroke or its occurrence in patients detected at risk by transcranial Doppler. Except for acute anemic episodes such as acute splenic sequestrations or Parvovirus-related erythroblatopenia for which simple red blood cell transfusion is required, manual or automated exchange-transfusion is preferred, resulting in the best decrease in hemoglobin S level without increasing viscosity. 

Major drawbacks of transfusions are the risk of iron overload, alloimmunization, and delayed hemolytic transfusion reaction [49]. The risk of viral contamination has been drastically reduced in high-income countries, but remains a concern in low-income countries where viral detection and filtration of blood are not always available. The risk of alloimmunization is exacerbated in the US and Europe by the fact that the blood donor population is often genetically different to that of sickle cell patients. To prevent this risk, extended erythrocyte phenotyping of the patient’s blood is recommended at diagnosis for all sickle cell patients, and phenotype matching should be done for all transfusions. The risk is known to be higher for transfusions performed during acute crises than during scheduled monthly transfusions [49]. Iron overlad can be controlled by efficient but costly oral chelation [8]. The cost of chronic transfusion with chelation has been estimated to be approximately $40,000/year [50], but a stroke requires rehabilitation costs of $40,000/year in addition to the cost of life-long transfusions with chelation [51]. Moreover, long-term chronic transfusion often requires adequate central venous access. 

### 2.2. Stem Cell Transplantation

#### 2.2.1. Transplantations from an HLA-Identical Sibling (Matched-Sibling Donor) 

In 2017, an international survey reported the results from 1000 transplantations with sibling donors [16]. The majority of transplants were done in children younger than 16 years of age (*n* = 846), 90% of whom had been prepared with a myeloablative conditoning regimen. Events were defined as death, and early or late rejection. In children, overall survival was 95% (95% CI, 93–97%) and event-free survival (EFS) was 81% (95% CI, 74–88%) with a 13.3% (95% CI, 10.9–16%) risk of chronic-graft-vs-host disease (GVHD) [13]. 

In France, between 1988 and 2012, 234 patients younger than 30 years, 202 being younger than 15 years and 32 being older (15–30 years) were transplanted after a homogeneous myeloablative conditioning regimen consisting of the association of busulfan and cyclophosphamide with rabbit ATG (Genzyme, 20 mg/kg). Rejection was defined as less than 5% donor cells. The first report on 87 patients transplanted between 1998 and 2004 [15] had shown a significant improvement with time, with 95.3% EFS (defined here as survival without TRM or rejection) for the last 44 patients transplanted since year 2000. These excellent results were confirmed with a 5-year EFS of 97.8% (95% CI: 95.6–100%) for the 190 patients (Jan-2000–Dec-2012) prepared with ATG, with 0.7% (95% CI: 0–2.1%) transplant-related mortality (TRM) and 1.5% rejection (95% CI: 0–3.7%) [17]. In this cohort, a significant difference was observed in the 5-year chronic-GVHD according to age at transplant with 7.6% occurrence (95% CI: 3.8–11.4%) in children younger than 15 years vs 29.7% (95% CI: 13.1–46.3%) in older ones. Low ATG dose and donor’s age were independent risk factors for chronic-GVHD. No significant EFS difference at five years was observed in bone marrow (BM) vs cord blood (CB) transplantation, but there was a significant higher risk of non-engraftment after CBT vs BMT (*p* = 0.017) and a trend to lower mortality rate after CBT [17]. Mixed chimerism, defined as <95% donor cells, was frequent, observed in 44% of patients at one-year, but no crisis occurred in those with >15% donor cells, while some hemolysis stigmata were seen in those with less than 50% of donor cells [17].

In the US, interesting results in adults were obtained in two centers (Bethesda and Chicago) using the NIH non-myeloablative conditioning, i.e., total body irradiation (TBI) 3Gy and alemtuzumab 1 mg/kg, associated with a GVHD prophylaxis by sirolimus known to facilitate the tolerance [18,19,20]. EFS was 87%, with a rate of rejection of 13%, but there was no TRM or GVHD in these two series (*n* = 43). Only one death occurred in one patient who had rejected the graft and had severe Moya. The same protocol was recently and successfully used in 14 children in Calgary, Canada [52]. However, there were some concerns about using TBI in children and the prolonged immunosuppression needed with this protocol.

#### 2.2.2. Unrelated Stem Cell Transplantations

The Sickle Cell Unrelated donor Transplant SCURT trial using a reduced intensity conditioning (RIC) and unrelated CB was prematurely stopped because of the occurrence of five rejections among the eight children transplanted and two GVHD with one fatal extensive GVHD [23]. The same protocol as the one in the SCURT trial was applied to 29 children for unrelated BMT. The primary endpoint of 75% EFS at one year was reached, but the rate of chronic GVHD was high, with 38% extensive GVHD [23]. In these trials, alemtuzumab was given between day 22 and day 18 before graft infusion in order to prevent rejection; however, better GVHD prevention might be obtained if given later in the conditioning regimen.

#### 2.2.3. Related Haplo-Identical Transplantations

The number of haplo-SCT performed in children for SCD remains limited, but the results are promising. The John Hopkins protocol using post-transplant cyclophosphamide in 17 patients older than 15 years resulted in 50% rejection risk, but no GVHD and no TRM [21]. Despite the excessively high rate of rejection, this first report was of importance as almost all SCD-patients had a related-haplo-identical donor and it seemed very interesting to be able to stop chronic transfusion in one-half of severe SCD-patients [53]. In order to reduce the rejection rate, the protocol was modified by adding Thiotepa in the conditionning [54]. This was used in adults and in 6 children and resulted in a significant improvement of engraftment with only 6.7% rejection, no TRM, 93% EFS, but still 7% chronic-GVHD, although lower than with unrelated transplants. An another protocol using CD3+/CD19+ depletion has shown interesting results in 6 SCD-children, with 83% EFS, but one death related to a fatal CMV pneumonitis [55]. 

## 3. Who to Consider for Allogeneic Stem Cell Transplantation in Children with Sickle Cell Disease?

### 3.1. Cerebral Vasculopathy

#### 3.1.1. Physiopathology

Cerebral vasculopathy is the most severe complication affecting children with SCA and its physiopathology is complex [56]. Traditionally, small vessel occlusion by intravascular sickling and sludging was considered responsible for strokes, but in the last 25 years, progressive major cerebral involvement has become recognized as the principal responsible factor. Inflammation, hypoxia, anemia, hemolysis and the resulting decrease in nitric oxide (NO) bioavailability impair blood rheology. Abnormal adhesion of sickle red blood cells and reticulocytes to the vasculature damages the vascular wall, causing intimal hyperplasia of large vessels, stenosis and progressive occlusion of the large cerebral arteries. Independent risk factors for abnormal TCD or stenosis occurrence are absence of associated alpha-thalassemia, severity of hemolytic anemia and G6PD deficiency [57].

Cerebral arteriopathy is progressive, promoting the development of a fine network of collaterals that can rupture and be responsible for hemorrhagic strokes. Thus, during infancy, strokes are most commonly ischemic, whereas after 20 years of age, hemorrhagic strokes are the most frequent [58]. Before TCD screening, the risk of stroke was 11% by the age of 18 [58] and occurred most frequently between five and 10 years of age. 

#### 3.1.2. Secondary Stroke Prevention

Chronic transfusion (CT) has been shown to reduce the risk of stroke recurrence from 67% to 10% [59], but stopping CT resulted in 50% risk of recurrence [60]. More recently, a 20% risk of stroke and silent infarct recurrence, and stenosis progression was reported on CT [61,62,63]. Hydroxyurea (HU) was proposed for secondary stroke prevention [64], but the SWiTCH trial comparing HU/phlebotomies to CT/oral chelation was prematurely stopped because of unacceptable occurrence of secondary strokes in the HU group (7/67 (10%) versus 0/66 in the CT group) [65]. In the literature, MSD-SCT has been reported in around 73 patients with stroke history. Results showed four hemorrhagic strokes, but no new ischemic stroke [13,14,15,66,67]. It is important to keep in mind that the risk of stroke persists in patients with Moyamoya, but it is still lower after SCT than on CT [68]. 

#### 3.1.3. Primary Stroke Prevention

##### Cerebral Vasculopathy Detection

Transcranial Doppler (TCD) is a non invasive technique measuring the flow velocities in the middle cerebral, anterior and internal carotid arteries via a temporal window [9,10]. Cerebral velocities increase until five years of age and decrease thereafter, and are higher in SS/Sβ0-Thal (SCA) than in SC/Sβ+Thalpatients [28].

More recently, the extracranial part of the internal carotid has been shown to be affected by arteriopathy in SCA [69,70,71,72,73]. Thus, the assessment of the extracranial part of the internal carotid has been added by color Doppler using a submandibular approach [70,73] and by cervical magnetic resonance angiography (MRA) [72,73].

Time averaged mean of maximum velocities are used to classify the velocities as abnormal when they are equal to or higher than 200 cm/s, with a 40% stroke risk within 36 months, conditional when between 170 and 199 cm/s, with a 7% stroke risk, and normal when lower than 170, which is associated with only a 2% of stroke risk [9,74]. In the Créteil newborn cohort, the cumulative incidence of abnormal-TCD was approximately 30% by the age of nine and reached a plateau thereafter [28].

##### Primary Stroke Prevention by Chronic Transfusion

The randomized STOP-1 trial compared CT to simple observation, and showed a drastic reduction of stroke risk (92% difference, *p* < 0.001) on CT [75]. The randomized STOP-2 trial posed the question of the required duration of CT and compared pursuing CT to stopping it in patients who had normalized velocities on CT for at least 30 months and no severe stenosis. The results showed a high rate of stroke and abnormal-TCD recurrence after discontinuation of CT [76]. The results of the STOP-2 and the high number of SCA-patients with a risk of abnormal-TCD [28] led to evaluating the effects of hydroxyurea and SCT in order to avoid long-term CT that has high risk of alloimmunization and iron overload [77,78]. 

##### Primary Stroke Prevention by Hydroxyurea

At the Créteil Center (France), since 1998, patients with normalized velocities and no stenosis were switched to HU, with a trimestrial control of the TCD and immediate reintroduction of CT in case of reversion to abnormal-TCD [77]. No stroke was observed, but TCD reversions and CT reinitiation occurred in about one third of the patients [66]. In the US and Canada, a randomized, non-inferiority trial was run between 2011 and 2013 after at least 12 m CT in patients with no severe vasculopathy [78]. Non-inferiority was met, but it is important to underscore that the follow-up was short with only 50% of the patients reaching two-year follow-up, the mean duration of CT before enrollment was long and the mean age at enrollment was 9.7 years, so most patients might have not have been at risk at the time of enrollment [79]. Thus, a longer follow-up will be required to ascertain that switching to HU is definitely safe. 

##### Primary Stroke Prevention by Stem Cell Transplantation

**Figure 1:**
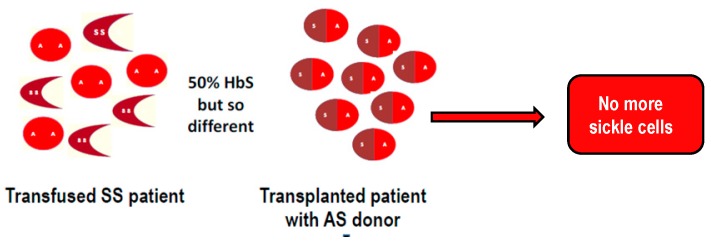
Difference between a transfused sickle-cell anemia (SCA)-patient who has a double population of sickle SS cells and transfused normal AA red cells and a transplanted patient who has only normal red blood cells as the donor (AS or AA) and no more sickle cells.

The DREPAGREFFE prospective trial comparing MSD-SCT to standard care in patients on long-term chronic transfusion for abnormal-TCD has been recently reported [80]. To be included, SCA-children had to be five to 15 years old, have at least one non-SCA sibling with parents agreeing to familial HLA-typing and SCT if available MSD. In the standard care group, CT was maintained for at least one year, before switching to hydroxyurea in those with normalized velocities and no stenosis. Between Dec-2010 and June-2013, 67 children were enrolled (seven of them had a stroke-history), 32 with a MSD were transplanted and 35 maintained on CT with a three-year follow-up. No death or stroke was observed in either group. No rejection or chronic GVH was observed in the transplantation group,. Velocities were highly significantly lower in the transplantation than in the standard care group at 1- and 3-year follow-up (129.6 cm/s vs. 170.4 cm/s, difference −40.8 cm/s, *p* < 0.001 at 1 year and 112.4 cm/s vs. 156.7 cm/s, difference −44.3 cm/s, *p* < 0.001, at three years). This could be explained by the fact that with CT, patients have a double population of red cells with normal AA and SS, whereas after SCT, even in those transplanted with and AS donor, sickle cells are no longer present (Figure 1) [80]. Moreover, the proportion of patients with normalized velocities (<170 cm/s) was significantly higher after SCT than on standard-care (80% vs. 48% at 1 year; *p* = 0.045).

#### 3.1.4. Silent Cerebral Infarcts

##### Detection, Prevalence and Risk Factors

Silent cerebral infarcts, defined as ischemic lesions on magnetic resonance imaging (MRI) in absence of clinical cerebral event, have been shown to be associated with significant cognitive impairment [81,82,83]. They can be progressive [84] and are associated with risk of subsequent overt stroke [85]. In a newborn cohort, despite early TCD screening the cumulative incidence was 37.7% by age 14 with no plateau [28]. The absence of plateau was confirmed in adult patients with a 53% prevalence by age 30 [86]. Risk factors for silent cerebral infarct occurrence are low baseline hemoglobin level [28,87], relative hypertension, and male gender [87], presence of intra [28,88], and extracranial [89] stenosis, acute and chronic anemia [89]. 

##### Prevention of Silent Cerebral Infarcts by Chronic Transfusion

In SCA-children with silent cerebral infarcts and no abnormal TCD, a three-year randomized trial compared the occurrence of strokes, new cerebral silent infarcts and enlargement of pre-existing silent infarcts in patients maintained on observation vs those placed on CT [90]. There was a significantly lower stroke and silent infarct incidence on CT (2.0 vs. 4.8/100 patient-years on simple observation; *p* = 0.04) [90], but the difference between CT and observation was less significant that seen in the STOP-1 trial in patients with abnormal-TCD in which stroke incidence was reduced by 92% (*p* < 0.001) [65]. 

##### Prevention of Silent Cerebral Infarcts by Hydroxyurea

The impact of hydroxyurea on silent infarct occurrence or recurrence has not been tested in randomized trials. While the improvement of anemia on hydroxyurea could suggest some silent infarct prevention, several observational studies have reported a persistant incidence of silent infarct on hydroxyurea therapy [91,92]. 

##### Prevention of Silent Cerebral Infarcts by Stem Cell Transplantation

Children successfully engrafted after SCT had no new silent infarct occurrence on MRI [14,15,66,80]. Thus, SCT offers the best prevention for silent cerebral infarcts.

#### 3.1.5. Cognitive deficiency

Cognitive performances in children and adults with SCA have been reported to be lower [93,94,95,96,97] than in healthy controls or non-SCA siblings and seem to decrease during aging [98]. Silent infarcts have been found to be associated with lower performances [94], while another large study found that independent risk factors associated with cognitive deficiency were the presence of ischemic lesions on MRI, severe anemia and thrombocytosis [95]. The Silent Cerebral Infarct Multi-Center Clinical Trial (SIT) comparing chronic transfusion to simple observation did not find significant difference in cognitive performances between both groups during the 3-year follow-up [90]. Hydroxyurea, which improves anemia, could be useful to protect cognitive performances. However, to date, no significant improvement has been shown in randomized trials. In the Baby-Hug trial comparing hydroxyurea to placebo, no difference was found between groups and only a trend toward better cognitive performances was reported in those on hydroxyurea with baseline severe anemia [99]. Following SCT, stabilization with age of cognitive performances has been found [100], but the DREPAGREFFE trial comparing chronic transfusion to HSCT in children with abnormal-TCD history did not find any significant difference in cognitive performances between both groups at the 1 and 3-year follow-up. However, a significantly higher progression of the processing speed index was seen in the transplantation group from 1 to 3 years : 6.1 (12.9) vs. −3.1 (10.5) in the standard-care group, *p* = 0.02 [80].

### 3.2. Prevention of Vaso-Occlusive Crisis and Acute Chest Syndrome

Pain and acute chest syndromes (ACS) were significantly reduced on CT with 42 pain crises/100 patient-years vs. 102 pain crises/100 patient-years on observation, and 1.8 ACS/100 patient-years vs. 14.3/100 patient-years on observation [90]. Similar results were obtained with hydroxyurea therapy. In the Baby-HUG trial, 94 pain crises/100 patients-years occurred on hydroxyurea vs. 203/100 patient-years on placebo [101], and 4.2 ACS/100 patient-years vs. 14.6/100 patient-years on placebo [101].

In transplanted children, no more vaso-occlusive crisis and ACS were observed in successfully engrafted patients [11,12,13,14,15,67,80]. This is different in adults where more time post-transplant was required for pain control [19].

### 3.3. Spleen Function

Functional asplenia occurs early in the course of the disease as the result of vaso-occlusive events occurring in the spleen. The high viscosity of sickle-cell blood causes a diversion of splenic blood flow through intrasplenic shunts, thus bypassing the phagocytic reticuloendothelial elements of the organ. This loss of reticuloendothelial function is responsible for the presence of Howell-Jolly bodies on blood smears and absence of technetium 99 m splenic uptake. When sickled cells were replaced by normal red cells through transfusion, splenic circulation and function were temporarily restored [102]. The Baby-Hug trial randomized hydroxyurea at 20 mg/kg/day versus placebo in very young children (nine to 18 months old at enrollment) and splenic function was not significantly different between both groups [36], but it was found to be improved in another study using hydroxyurea at the maximal tolerated dose [103]. However, after HSCT, the number of Howell-Jolly bodies decreased or permanently disappeared with normalization of splenic uptake, at least in patients transplanted during infancy [12,104,105], showing that this organ damage previously considered as permanent can in fact be reversed after HSCT in children with SCA.

### 3.4. Kidney Function

Glomerular involvement is characterized by an early increase in glomerular filtration rate (GFR), followed by a gradual decline of GFR and chronic renal failure [106,107,108]. Microalbuminuria, with or without hyperfiltration, is currently the earliest renal symptom detectable, reflecting probable glomerular injury in adults and children with SCD [109]. The prevalence of albuminuria in SCD patients increases with age, from 4.5% to 26% in patients under the age of 21 years to between 26% and 68% in older patients [109]. Long-term outcome of kidney function after intensive therapies such as 6-month hydroxyurea, CT or HSCT are currently missing. In the Baby-Hug trial, GFR was not different in children treated with hydroxyurea than in the placebo group [36]. A pilot study in adults suggested that hydroxyurea significantly decreased microalbuminuria [110]. After HSCT, stabilization was observed in four adult patients [19]. 

### 3.5. Pulmonary Function

Progressive severe restrictive syndrome has been observed in adult SCA-patients [111]. HSCT may be toxic for pulmonary function because of the use of busulfan and the risk of chronic GVHD-related obliterans bronchiolitis [112]. However, except in patients with obliterans bronchiolitis, pre- and post-HSCT pulmonary function tests showed stable total lung capacity and improvement of carbon monoxide diffusing capacity [113]. Tricuspid regurgitant jet velocity (TRJV) associated with NT-proBNP and the 6-minute walk test are useful to detect patients at risk of pulmonary hypertension, which is mainly diagnosed as a mean pulmonary artery pressure (mPAP) > 25 mm Hg by right heart catheterization; however, TRJV > 2.5 m/s was shown to a be a risk factor for early death [114]. Pulmonary hypertension is very rare in SCA-children but a significant decrease of TRJV was observed in adult patients after HSCT [19]. 

### 3.6. Gonadal Function

In boys, puberty was normal after SCT, with normal levels of testosterone, follicle stimulating hormone (FSH) and luteinizing hormone (LH) [17,113]. It is well established that Sertoli cells responsible for spermatogenesis are more easily damaged than Leydig cells, and that testicular size and semen analysis better predict gonadal function and fertility potential. However, no post-SCT semen analysis was performed [17] but it would also be critical to compare these results to those obtained in SCD patients who did not receive transplants and who frequently have oligoazoospermia [115,116]. In the French series [17], three males among 19 males older than 25 years spontaneously fathered children 11 to 20 years post-HSCT. Nevertheless, pre-transplant sperm cryopreservation should be performed in all pubertal males and pre-transplant testicular tissue cryopreservation is recommended in the prepubertal ones [17]. 

In contrast, all girls who were post-pubertal at the time of transplantation developed amenorrhea with low serum estradiol and elevated LH and FSH levels during the year following transplant, necessitating hormone replacement therapy [17]. Most of those who were prepubertal required hormonal substitution for puberty induction. However, normal puberty often occurred spontaneously in the girls transplanted earlier at the mean (SD) age of 5.9 (2.6) vs. 10.1 (2.1) (*p* = 0.002) [17]. Spontaneous pregnancies were observed in four females transplanted approximately 20 years ago and at young age (5.8–7.7 years) showing that some ovarian function restoration is possible after a long time post-HSCT and in girls transplanted at young age. Moreover, two normal births occurred after orthotopic ovarian fragments autograft in a female whose one ovary had been cryopreserved [17]. Thus, unilateral oophorectomy should be recommended for ovarian cortical fragments cryopreservation [17] before HSCT using myeloablative conditionings. 

### 3.7. Growth 

Growth after HSCT is always normal in boys. It was also normal in girls, except in those with pubertal delay related to hormonal deficiency. However, after hormonal substitution, growth became normal [113]. 

### 3.8. Osteonecrosis

SCD is the most common cause of osteonecrosis of the femoral head during chilhood. Osteonecrosis is induced by a temporary or permanent loss of blood supply to the bone. Its natural history in SCD is poor, with progression to femoral head collapse and deformity. After HSCT, osteonecrosis can also occur in patients treated with steroids because of GVHD. In SCD-patients, an interesting approach using percutaneous implantation of autologous bone marrow osteoprogenitor cells has been successfully developed at early stages of osteonecrosis, allowing to decrease pain and delay the progression of the disease and postpone the need for hip arthroplasty [117,118]. Thus, we suspect that HSCT might be useful to improve osteonecrosis in transplanted patients with no GVHD. This was the case the first patient reported in 1997 [119]. Since this observation, several other cases had favorable outcome, but have not been reported. It would be important to compare this outcome to that of non-transplanted SCD-children.

### 3.9. Health-Related Quality of Life

Health-related quality of life (HRQL) is poor in SCA patients, even lower than in children treated for cancer [120].

The impact of hydroxyurea was first reported in the first randomized trial comparing hydroxyurea to placebo in adults. After over two years of treatment, hydroxyurea treatment improved HRQL only in patients who maintained a high HbF response compared to those with low HbF response or on placebo [121]. A cross-sectional retrospective study in children showed that the total score of HRQLwas 6 points better on hydroxyurea than without treatment [122]. Another cross-sectional study in 34 young SCD-patients (range: 12–18 years) showed a significant positive correlation of HRQL scores with HbF level and MCV and a negative correlation with fatigue and social isolation scores. Low HRQL scores and low HbF levels were associated with low adherence to hydroxyurea in 75% of these young patients [123]. 

The impact of chronic transfusion on HRQL was reported in the randomized SITT trial, comparing CT to simple observation. Physical functioning was 11.5 points better on CT than on simple observation [124]. 

After SCT, a significant improvement of overall HRQL was observed [125,126]. In 17 SCA-patients younger than 21 at transplant time, HRQL, was 16.6 points better at 1-year post-transplant than before transplant [125]. Interestingly, even in the trial of unrelated HSCT [23] in 30 children with SCD in which the 1-year rate of extensive GVHD was very high (38%), a significant improvement of HRQL was observed at 1-year post-HSCT [23]. At 1 year, transplanted children in the DREPAGREFFE prospective trial [80] reported significantly better physical functioning (*p* = 0.04) with lower pain rate (*p* = 0.009), higher energy (0.002) and better school functioning (*p* = 0.02) with lower numbers of days missed in school than transfused children. At 3-years, transplanted children not only reported better physical functioning than standard-care children (*p* = 0.001), but also less difficulties walking, running, participating in sports and things age-matched peers can do, less trouble sleeping (*p* = 0.05), and worrying less about the future (0.01). Total score HRQL at 3 years was 84.8 post-transplant vs 73.2 on standard care (*p* = 0.002), showing that MSD-SCT offers a better HRQL than CT or hydroxyurea [80]. Thus, a significant improvement of HRQL was obtained after SCT, which was higher than with hydroxyurea or chronic transfusion. This is another factor that families and caregivers should consider before deciding upon SCT in patients with SCD. 

## 4. When to Consider Allogeneic Stem Cell Transplantation in Children with Sickle Cell Disease?

**Figure 2:**
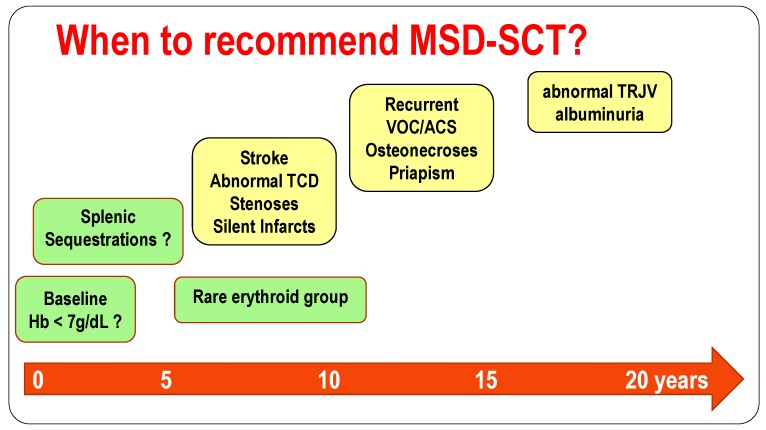
When to recommend SCT in children with sickle cell disease?

In absence of consistent genetic markers detectable at birth that can predict the severity of the sickle cell anemia, and considering the low but not nul risk of early death post-transplant, GVHD and infertility risks, SCT should be first discussed with parents and providers when clinical and biological complications start appearing during infancy. Strokes, stenoses, silent strokes associated with cognitive deficiencies, or frequent VOC/ACS despite hydroxyurea are well-accepted indications for SCT (Figure 2). 

However, SCT might also be discussed in patients experiencing splenic sequestrations that require CT during at least 2 years followed by splenectomy, and in our experience, are associated with subsequent higher rate of crises [127]. SCT should definitely be discussed before deciding on splenectomy, as a significant improvement of splenic function has been shown post-SCT [12,15,104,105]. It should similarly be discussed when patients have rare erythroid groups, resulting in the risk of transfusional impasse. Low baseline hemoglobin level (Hb < 7 g/dL) is now recognized as being associated with many SCD-related complications such as strokes [58], abnormal-TCD [28,57,73], silent infarcts [28,73,87,89], cognitive deficiency [82,83], kidney failure [128], pulmonary hypertension [129] and early death [30], and SCT should be discussed in each of those cases. Abnormal TRJV associated with a risk of early death [114] and kidney complications [106,107,108,109,110,128] such as permanent microalbuminuria and albuminuria should be indications for SCT before organ failure occurs.

Neverthless, several genetic markers have been shown to predict the severity of the disease and could help to identify children at very high risk for whom SCT could be an early recommendation. The presence of alpha-thalassemia, which is associated with a higher level of hemoglobin and lower risk of stroke [130] and abnormal-TCD [57], may be associated with a higher rate of crises [131]. A low level of HbF at baseline is associated with the risk of frequent VOC or ACS and early death [30] and with CAR beta-haplotype. In contrast, BEN, SEN and Arab-Indian beta-haplotypes are associated with higher level of HbF and better prognostic. The most severely anemic patients have the CAR/CAR beta-haplotype with no alpha-thalassemia [41]. BEN/BEN patients have a higher prevalence of the favorable *BCL11A/*rs1427407 T/allele and better response to hydroxyurea than CAR/CAR patients [41]. The most severe SCA-patients live in Central African countries where the CAR haplotype is predominant, but resources for treatment, particularly SCT, are largely missing [3]. However, it is important to keep in mind that SCT is in fact cost-effective [132,133]. The first successful MSD-SCT for SCD occurred in Nigeria [134], which to date remains the only African country having this technology available.

## 5. How? Choice of Conditioning Regimen and Transplantation Types as a Function of Donor Availability and Disease State

### 5.1. Cerebral Vasculopathy

Based on the studies so far, patients with stroke history and/or stenoses should be transplanted with MSD if available or with haplo or unrelated donor. Those with abnormal TCD placed on CT should be transplanted with MSD. In absence of MSD, chronic transfusion should be pursued and we recommend to introduce hydroxyurea in those with normalized velocites and no stenosis, but to consider haplo and unrelated SCT for those with stenoses.

Patients with cerebral silent infarcts but no stenosis and with MSD should be transplanted while haplo and unrelated SCT should only be recommended in those who develop new silent infarcts or enlargement of silent infarcts on hydroxyurea.

**Figure 3:**
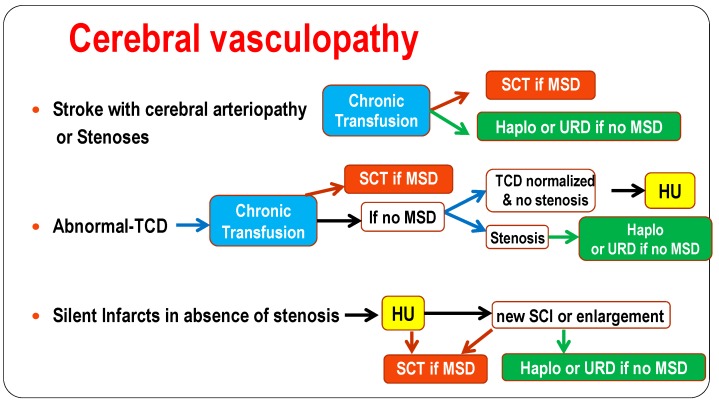
Indications of the different treatments: hydroxyurea, chronic transfusion and. type of SCT to recommend in children with cerebral vasculopathy. Abbreviations: HU = hydroxyurea, MSD = matched-sibling donor, SCT = stem cell transplantation, Haplo = related haplo-identical, URD = unrelated stem cell-transplantation.

However, we have to keep in mind, that early toxicity occurs frequently after HSCT for SCD. Seizures and posterior reversible encephalopathy syndromes (PRES) may occur despite preventive measures such as anticonvulsant prophylaxis with Clonazepam during busulfan administration and cyclosporine therapy, strict control of arterial hypertension, prompt correction of magnesium deficiency, maintenance of the hemoglobin concentration above 9 g/dL and platelet count above 50,000 per cubic millimeter [15,17,135,136]. They occur independently of the pre-transplant presence or absence of cerebral vasculopathy, and are associated with cyclosporine, hypertension and steroids [15] (Figure 3). This risk has been significantly reduced by promptly replacing cyclosporine with mycophenolate mofetil in case of GVHD requiring steroid therapy [17]. However, the risk of seizures and PRES remains an adverse effect of cyclosporine and steroid therapy, and may warrant substituting cyclosporine with sirolimus [18,19] that is less neurotoxic and is a good inducer of tolerance [137].

### 5.2. Crises Vaso-Occlusives (CVO) and/or Acute Chest Syndromes (ACS)

**Figure 4:**
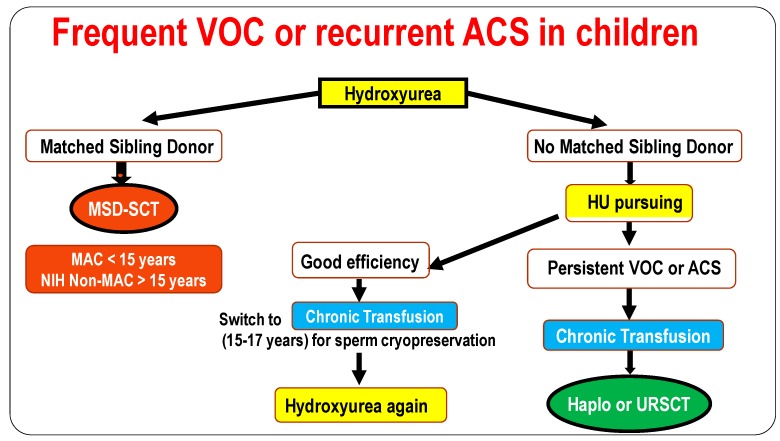
Indications of the different treatments: hydroxyurea, chronic transfusion and. which kind of stem cell transplantation to propose in children with frequent vaso-occlusives or recurrent acute chest syndrome Abbreviations: VOC = vaso-occlusive crisis, ACS = acute-chest syndrome, HU = hydroxyurea, MSD = matched-sibling donor, SCT = stem cell transplantation, Haplo = related haplo-identical, URD = unrelated stem cell-transplantation, MAC = myeloablative conditioning.

Hydroxyurea is the treatment of choice for children experiencing frequent VOC or ACS (Figure 4). However, because of the frequent issue with treatment adherence and the fears of infertility in males [44], familial HLA typing should be performed and SCT recommended mainly for those with a MSD. For those without MSD and still experiencing frequent VOC or ACS on hydroxyurea, CT should be recommended first and haplo-identical SCT be considered. In those for whom hydroxyurea is quite efficient, transitory CT could be recommended between the age of 15–17 years in order to cryopreserve their sperm. 

### 5.3. Children with MSD: Choice between Myeloablative or Non-Myeloablative Conditioning Regimen?

The main issue in children with a MSD is to choose between myeloablative conditioning regimen, which results in 98% EFS, but 6% chronic GVHD risk before 15 years of age and fertility risk [17], and the NIH non-myeloablative conditioning regimen with 13% rejection, 87% EFS, no GVHD and no TRM, but longer immunosuppression required to obtain tolerance [18,19,20]. In France, we have opted for myeloablative conditioning for children younger than 15 years and the NIH non-myeloablative for older patients. However, this recommendation could change when results of long-term follow-up after non-myeloablative conditioning are available, as these have remained limited until now.

### 5.4. Children without MSD: Choice between Unrelated Transplantation or Related-Haplo-Identical?

Major concerns with unrelated-SCT for children with SCD are the low chance of finding a good donor among Afro-American children and the almost nul one for those from Central-Africa, and the overly high risk of GVHD (38% extensive) reported in the SCURT-trial [23], even if better results could be obtained with different conditioning regimens.

The major interest in related-haplo-identical SCT for SCD is that almost all SCD-patients have a donor (two parents and one-half of non-SCA siblings). The major concern has been the high rate of rejection in the first report [21], but progress has been made in reducing the rate of rejection by adding thiotepa [54] or by increasing the TBI to 4Gy [138] in the conditioning regimen. The risk of GVHD is not nul, but significantly lower than with unrelated-SCT.

Thus, haplo-SCT should be preferred to unrelated-SCT in SCD-patients without MSD. These two modalities of SCT should be limited to those with very severe disease such as patients with stroke history or stenoses and those who experience new or progressive cerebral silent infarcts or frequent crises or severe ACS despite intensive therapy. Moreover, haplo-SCT should also be discussed in those with severe erythroid alloimmunization, but should be only performed in controlled experimental studies.

### 5.5. Children without MSD: is Gene Therapy in the Near Future? 

Following the first successful results obtained in transfusion-dependent thalassemic patients with lentiviral β^A(T87Q)^-globin gene transfer [139,140], the first sickle cell patient experiencing very frequent complications was successfully treated with the same β^A(T87Q)^-globin gene transfer, which has an anti-sickling effect [141]. Thereafter, other sickle cell patients were treated in the US. After disappointing results in the first seven patients, several changes in the protocols allowed to obtain a satisfactory median level of the therapeutic HbA^T87Q^ of 39% in the next six treated patients [142]. Thus, these results are truly promising and probably will allow the disappearance of vaso-occlusive crises and most of sickle cell disease-related complications. For cerebral vasculopathy, which requires high levels of HbA to control the disease, this current technology may not successfully reach this goal. In addition, it is important also to remember that gene therapy requires myeloablative conditioning using high doses of busulfan that increases the known risk of infertility, although with no GVHD risk. Moreover, to date, this treatment is only accessible to few selected patients. If longer follow-up confirms these excellent results and the safety of the procedure, gene therapy could be applied to many patients having no MSD and will have to be compared with the results of haplo-transplant.

## 6. Summary

Sickle cell anemia remains a very severe disease compromising socio-professional insertion and quality of life, but stem cell transplantation offers a cure. If a matched-sibling donor is available, it should be recommended as soon as intensive therapies such as hydroxyurea or chronic transfusion are required to treat or prevent complications in these children. 

In absence of MSD, alternative SCT should be discussed and offered to patients with severe complications despite standard treatments, and related-haplo-identical SCT should be preferred to unrelated transplantations to limit GVHD risk. Finally, gene therapy should be considered when successful results and safety are confirmed. 

Until now, myeloablative conditioning regimens have given the best chance of cure in children younger than 15 years, but require cryopreserving of ovarian or testis to alleviate possible sterility. After the age of 15 years, despite a higher risk of rejection, non-myeloablative such as the NIH-protocol could be preferred in order to avoid GVHD. Neverthless, it will be very important to compare the long-term outcomes after HSCT according to conditioning regimens and to SCD alternative therapies such as chronic transfusions, hydroxyurea or gene therapy.

Finally, as low adherence to treatment drugs such as hydroxyurea taken throughout life is frequent in SCD patients, the highly significant improvement of HRQL post-SCT should be stressed to patients, parents and caregivers, which might help them to decide on HSCT that can cure the disease.

These recommendations are only applicable in countries with high income resources; nevertheless, they should be reconsidered in African countries where the majority of patients live and where the chances of having a matched-sibling donor are higher. In order to limit the extraordinary heavy burden of this disease worldwide, it would be crucial to detect sickle cell trait in young adults, to perform genetic counseling, to offer access to efficient drugs such as hydroxyurea to sickle cell children, and to develop “exportable” techniques, both for the selection of patients (TCD, family HLA typing etc.,) and for the transplant itself.
